# A Nonsynonymous Polymorphism in *Semaphorin 3A* as a Risk Factor for Human Unexplained Cardiac Arrest with Documented Ventricular Fibrillation

**DOI:** 10.1371/journal.pgen.1003364

**Published:** 2013-04-11

**Authors:** Yukiko Nakano, Kazuaki Chayama, Hidenori Ochi, Masaaki Toshishige, Yasufumi Hayashida, Daiki Miki, C. Nelson Hayes, Hidekazu Suzuki, Takehito Tokuyama, Noboru Oda, Kazuyoshi Suenari, Yuko Uchimura-Makita, Kenta Kajihara, Akinori Sairaku, Chikaaki Motoda, Mai Fujiwara, Yoshikazu Watanabe, Yukihiko Yoshida, Kimie Ohkubo, Ichiro Watanabe, Akihiko Nogami, Kanae Hasegawa, Hiroshi Watanabe, Naoto Endo, Takeshi Aiba, Wataru Shimizu, Seiko Ohno, Minoru Horie, Koji Arihiro, Satoshi Tashiro, Naomasa Makita, Yasuki Kihara

**Affiliations:** 1 Department of Cardiovascular Medicine, Division of Frontier Medical Science, Programs for Biomedical Research, Graduate School of Biomedical Science, Hiroshima University, Hiroshima, Japan; 2 Laboratory for Digestive Diseases, Center for Genomic Medicine, RIKEN, Hiroshima, Japan; 3 Department of Gastroenterology and Metabolism, Division of Frontier Medical Science, Programs for Biomedical Research, Graduate School of Biomedical Science, Hiroshima University, Hiroshima, Japan; 4 Department of Cellular Biology, Research Institute for Radiation Biology and Medicine, Hiroshima University, Hiroshima, Japan; 5 Division of Cardiology, Nagoya Daini Red Cross Hospital, Nagoya, Japan; 6 Division of Cardiology, Department of Medicine, Nihon University School of Medicine, Tokyo, Japan; 7 Division of Heart Rhythm Management, Yokohama Rosai Hospital, Yokohama, Japan; 8 Department of Cardiovascular Biology and Medicine, Niigata University Graduate School of Medical and Dental Sciences, Niigata, Japan; 9 Division of Orthopedic Surgery, Niigata University Graduate School of Medical and Dental Sciences, Niigata, Japan; 10 Division of Arrhythmia and Electrophysiology, Department of Cardiovascular Medicine, National Cerebral and Cardiovascular Center, Suita, Japan; 11 Department of Cardiovascular Medicine, Shiga University of Medical Science, Otsu, Japan; 12 Department of Anatomical Pathology, Division of Frontier Medical Science, Programs for Biomedical Research Graduate School of Biomedical Science, Hiroshima University, Hiroshima, Japan; 13 Department of Molecular Physiology, Nagasaki University Graduate School of Biomedical Sciences, Nagasaki, Japan; University of Amsterdam, The Netherlands

## Abstract

Unexplained cardiac arrest (UCA) with documented ventricular fibrillation (VF) is a major cause of sudden cardiac death. Abnormal sympathetic innervations have been shown to be a trigger of ventricular fibrillation. Further, adequate expression of *SEMA3A* was reported to be critical for normal patterning of cardiac sympathetic innervation. We investigated the relevance of the semaphorin 3A (*SEMA3A*) gene located at chromosome 5 in the etiology of UCA. Eighty-three Japanese patients diagnosed with UCA and 2,958 healthy controls from two different geographic regions in Japan were enrolled. A nonsynonymous polymorphism (I334V, rs138694505A>G) in exon 10 of the *SEMA3A* gene identified through resequencing was significantly associated with UCA (combined P = 0.0004, OR 3.08, 95%CI 1.67–5.7). Overall, 15.7% of UCA patients carried the risk genotype G, whereas only 5.6% did in controls. In patients with *SEMA3A* ^I334V^, VF predominantly occurred at rest during the night. They showed sinus bradycardia, and their RR intervals on the 12-lead electrocardiography tended to be longer than those in patients without *SEMA3A* ^I334V^ (1031±111 ms versus 932±182 ms, P = 0.039). Immunofluorescence staining of cardiac biopsy specimens revealed that sympathetic nerves, which are absent in the subendocardial layer in normal hearts, extended to the subendocardial layer only in patients with *SEMA3A* ^I334V^. Functional analyses revealed that the axon-repelling and axon-collapsing activities of mutant *SEMA3A* ^I334V^ genes were significantly weaker than those of wild-type *SEMA3A* genes. A high incidence of *SEMA3A* ^I334V^ in UCA patients and inappropriate innervation patterning in their hearts implicate involvement of the *SEMA3A* gene in the pathogenesis of UCA.

## Author Summary

Unexplained cardiac arrest with documented ventricular fibrillation (UCA) is defined as spontaneous ventricular fibrillation (VF) that is not associated with known structural or electrical heart diseases and is one of the major causes of sudden cardiac death. Identification of the genes responsible for UCA may further increase our understanding of mechanisms of UCA and facilitate more accurate diagnosis and preventive treatment, especially in asymptomatic disease-carrying relatives of the patient. However, molecular mechanisms of UCA have not been fully clarified due to the high mortality rate and difficulty of diagnosis. In this study, UCA patients are shown to have a high incidence of a polymorphism in the Semaphorin 3A gene (rs138694505, *SEMA3A* ^I334V^). The result confirms previous reports that the abnormal sympathetic innervation is a trigger of UCA because *SEMA3A* is crucial for the establishment of normal innervation patterns in the heart. Furthermore, experimental data presented here indicate that *SEMA3A* ^I334V^ disrupts the *SEMA3A* function and impairs appropriate innervation patterning. Finally, the study suggests that *SEMA3A* ^I334V^ is a risk factor for human UCA and contributes to the etiology of UCA.

## Introduction

Unexpected sudden death in healthy individuals remains a daunting problem. Unexplained cardiac arrest with documented ventricular fibrillation (UCA) including idiopathic ventricular fibrillation (IVF) is defined as spontaneous VF that is not associated with a known structural or electrical heart disease. IVF is diagnosed in up to 10% of survivors of out-of-hospital cardiac arrest [1].

Many reports have documented the role of abnormal sympathetic innervations as a trigger of VF [2]–[6]. Sympathetic innervation of the heart is determined during development by chemoattractive and chemorepulsive factors. Semaphorins, members of a conserved family of both secreted and integral membrane proteins, are typical chemorepulsive factors acting on the growth cone as guidance cues to control the establishment of neural connections [7], [8]. Recently, *SEMA3A* was shown to form an epicardial-to-endocardial transmural sympathetic innervation pattern in the heart. In addition, disruption of innervation patterning in both *SEMA3A* -deficient and *SEMA3A*-overexpressing mice resulted in sudden death or lethal arrhythmias [9], [10].

Identification of the genes responsible for UCA may further increase our understanding of the pathophysiology of UCA and facilitate the diagnosis and prophylactic treatment, especially in asymptomatic, disease-carrying relatives of the proband. In the current study, we investigated the significance of the SEMA3A gene polymorphisms in the etiology of UCA.

## Results

### Genetic analysis of the *SEMA3A* gene in UCA patients

The subjects were divided into two geographic regions based on their birthplace information, as shown in Figure S1. The characteristics of the two regional groups of UCA patients enrolled in this study are listed in Table 1. There was no significant difference in the clinical characteristics among the two UCA groups. As for control groups, the gender distribution was similar in the two groups, but individuals were older in Eastern Japan as compared to Western Japan (70±9 years vs. 47±16 years).

**Table 1:** Characteristics of UCA with VF patients.

	Western Japan	Eastern Japan
No. of patients	52	31
Age (years)	43±17	43±14
Male gender	40 (76.9%)	24 (77.4%)
Documented VF	52 (100%)	31 (100%)
History of syncope	9 (17.9%)	9 (29.0%)
History of atrial fibrillation	11 (21.2%)	7 (22.6%)
Family history of sudden cardiac death	11 (21.2%)	6 (19.4%)
**Time of VF events**		
Daytime (8:00–20:00)	31 (59.6%)	17 (54.8%)
Nighttime (20:00–8:00)	21 (40.4%)	14 (45.2%)
**Situation at VF events**		
During exercise or physical effort	16 (30.8%)	11 (35.5%)
During sleeping or just after getting up	13 (25.0%)	13 (42.0%)
After meals or drinking	2 (3.8%)	1 (3.2%)
During driving	4 (7.7%)	1 (3.2%)
Relaxed at home	10 (19.2%)	2 (6.5%)
At restroom	2 (3.8%)	0 (0%)
Unknown/Others	9 (17.6%)	3 (9.6%)
**Posture at VF events**		
During standing	26 (50.0%)	13 (41.9%)
Seated or supine position	41 (46.2%)	17 (54.8%)
Unknown	2 (3.8%)	1 (3.2%)

UCA: unexplained cardiac arrest, VF:ventricular fibrillation, Data was mean±SD.

Only one nonsynonymous polymorphism was identified in exon 10 of the *SEMA3A* gene through resequencing of the coding region. This polymorphism causes an amino acid substitution from isoleucine to valine (I334V, *SEMA3A* ^I334V^) and is identical with the SNP that was recently submitted to dbSNP (rs138694505). There was a significant difference in genotype frequencies between UCA cases and controls in the western Japan (dominant model P = 0.007). This association was replicated in the Eastern Japan (P = 0.008). The Breslow-Day test showed no heterogeneity among the groups, and the overall degree of association by the Mantel-Haenszel test was P = 0.0004 (OR 3.08, 95%CI 1.67–5.70) (Table 2). Collectively, 13 of the 83 UCA patients (15.7%) carried the risk genotype G, whereas only 5.6% did in the controls. The *SEMA3A* ^I334V^ carrier frequency appeared to be relatively stable throughout the age classes (Data not shown).

**Table 2:** SEMA3A polymorphism (SEMA3AI334V: rs138694505) in patients with UCA and controls.

Region	IVF			control			Odds ratio	95%CI	P^a^	P_het_ b
	AA	AG	GG	AA	AG	GG				
Western Japan	45	7	0	1943	102	1	2.9	1.3–6.7	0.007	
	86.5%	13.5%	0.0%	95.0%	5.0%	0.0%				
Eastern Japan	25	6	0	850	60	2	3.3	1.3–8.3	0.008	
	80.6%	19.4%	0.0%	93.2%	6.6%	0.2%				
Combined^c^							3.08	1.67–5.70	0.0004	0.86

SEMA3A: semaphorin 3A, UCA: unexplained cardiac arrest,

a P value of chi-square test in dominant model,

b Result of Breslow-Day test,

c Combined meta-analysis was performed using the Mantel-Haenszel method.

### Genotype distribution of *SEMA3A* polymorphisms (rs138694505) among ethnicity

According to the 1000 Genomes Project, regional differences in the *SEMA3A* ^I334V^ (rs138694505) frequency are evident among populations. For example, the frequency of the G allele is 2.1% in East Asians (3.93% in Japanese), 1.35% in West Africans, 1.86% in Americans, and 0% in Europeans (Table 3).

**Table 3:** Regional differences in rs138694505 G allele frequency among populations.

Population			Genotype count				G allele frequency(%)
			AA	AG	GG		
European	CEU		87	0	0		0
	TSI		98	0	0		0
	GBR		89	0	0		0
	FIN		93	0	0		0
	IBS		14	0	0		0
	subtotal		381	0	0		0
East Asian	CHB		95	2	0		1.03
	JPT		82	7	0		3.93
	CHS		98	1	1		1.50
	subtotal		275	10	1		2.10
West African	YRI		85	3	0		1.70
	LWK		95	2	0		1.03
	subtotal		180	5	0		1.35
American	ASW		57	4	0		3.28
	MXL		64	2	0		1.52
	PUR		52	3	0		2.73
	CLM		60	0	0		0
	subtotal		233	9	0		1.86
Total			1069	24	1		1.19

Allele frequencies were estimated using the 1000 Genome Project dataset.

### Phenotype characterization of UCA patients and clinical findings of UCA patients with and without *SEMA3A* ^I334V^ (rs138694505)

**Figure 1:**
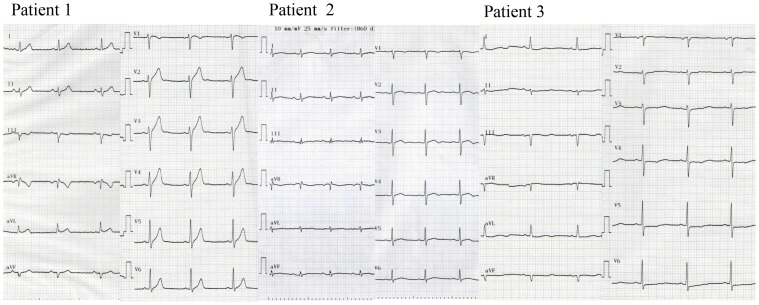
Twelve-lead ECG of patients with *SEMA3A* ^I334V^. Twelve-lead ECG of typical patients wth sinus bradycardia (left to right, each patient 1–3) before the ICD implantation.

**Figure 2:**
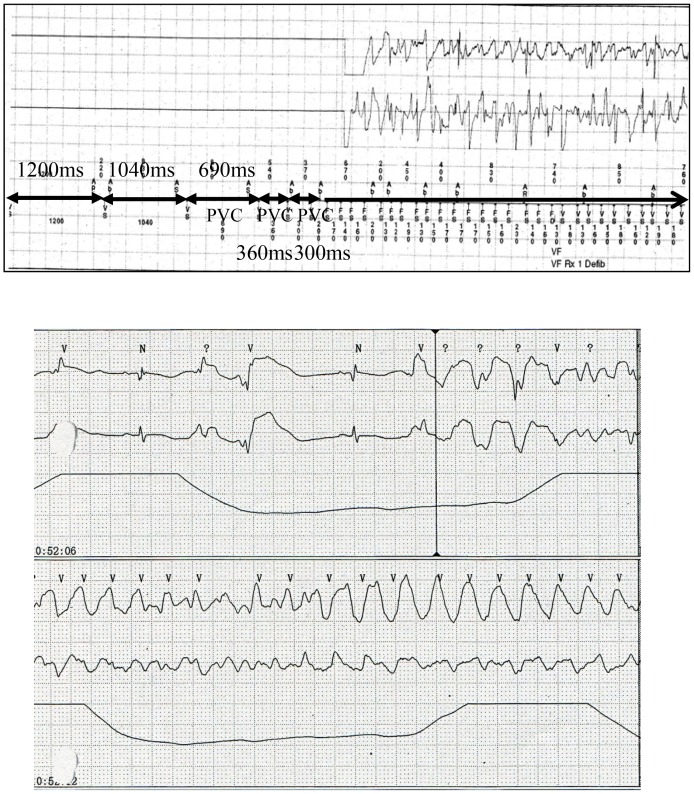
Ventricular fibrillation in patients with *SEMA3A* ^I334V^. After discharge, VF recurred twice and was terminated by ICD shocks in one male patient (patient 1). According to the ICD records, a preceding transient bradycardia was followed by short coupling ectopic ventricular beats and finally VF occurred (upper). The day after admission to the emergency unit, another female patient (patient 2) went into an electrical storm. VF occurred suddenly during sinus bradycardia (lower).

Two UCA cases were severe (Figure 1, patients 1 and 2). They suffered from VF at a young age and had a family history of sudden cardiac death. VF attacks recurred on several occasions in these patients. In one patient (Patient 1), VF recurred twice after discharge and was terminated by an implanted cardioverter defibrillator (ICD) shock (Figure 2 upper panel). According to the ICD records, a preceding transient bradycardia was followed by short coupled ectopic ventricular beats, finally leading to VF. Another patient (Patient 2) went into an electrical storm at midnight one day after hospitalization (Figure 2 lower). VF occurred suddenly during sinus bradycardia. She had been suffering repeated epileptic seizures with loss of consciousness from the age of 15. Most patients with *SEMA3A* ^I334V^ were found to have sinus bradycardia and sinus node dysfunction by an electrophysiological study. Figure 1 shows the ECGs before the ICD implantation in patients 1, 2 and 3. Because of the sinus bradycardia and in order to prevent a VF recurrence, the ICD was set to the AAI^+^ mode at 60–75 bpm. The number of tests that was performed in the UCA patients is shown in Table S2. The phenotype characterization in each UCA patient with *SEMA3A* ^I334V^ is shown in Table S3. Patient 3 had persistent AF and patient 13 had chronic AF. Patients 1,2,5,7 and 9 had 1st degree atrioventricular block.

In Table 4 we present the clinical, electrocardiographic, and echocardiographic findings between the UCA patients with and without *SEMA3A* ^I334V^. VF occurred predominantly at rest and during the night in the patients with *SEMA3A* ^I334V^. In contrast, it occurred during exercise and during the day in most patients without *SEMA3A* ^I334V^ (VF occurred during the night 69.2% vs. 37.1%, *P* = 0.032, VF occurred at rest 69.2% vs. 34.3%, *P* = 0.015).

**Table 4:** Comparison of the clinical and electrocardiographic findings in UCA patients with and without *SEMA3A* ^I334V^.

	*SEMA3A* ^I334V^: rs138694505 (+)	*SEMA3A* ^I334V^: rs138694505 (−)	
	N = 13	N = 70	
**Clinical Data**			
Age of VF occurrence (y)	48±17	42±16	P = 0.273
Gender (Male%)	9 (69.2%)	55 (78.5%)	P = 0.462
History of syncope	3 (23.1%)	15 (21.5%)	P = 0.894
History of atrial fibrillation	3 (23.1%)	15 (21.5%)	P = 0.895
Family History of SCD	4 (30.1%)	13 (18.5%)	P = 0.317
VF occurred during night time	9 (69.2%)	26 (37.1%)	P = 0.032*
VF occurred at rest	9 (69.2%)	24 (34.3%)	P = 0.015*
**Twelve Lead ECG Findings**			
RR (ms)	1031±111	932±182	P = 0.039*
PQ (ms)	180±36	171±30	P = 0.312
QRS (ms)	110±43	97±17	P = 0.498
QTc (ms)	421±35	413±42	P = 0.238
Presence of J wave	2 (15.4%)	34 (48.6%)	P = 0.020*
**Signal Averaged ECG Findings**			
fQRSd (ms)	128±31	121±24	P = 0.705
RMS 40 (uV)	39±28	30±26	P = 0.511
LAS 40 (ms)	36±7	37±10	P = 0.760
**Echocardiographic Findings**			
LVDd (mm)	46.5±5.2	48.1±5.7	P = 0.411
IVSTd (mm)	9.0±1.1	9.2±2.0	P = 0.881
EF (%)	66.2±6.9	63.4±7.6	P = 0.242

UCA: unexplained cardiac arrest, VF:ventricular fibrillation, SCD:sudden cardiac death, SEMA3A: Semaphorin 3A, LVDd: left ventricular end diastolic volume, IVSTd: interventricular septum thickness, EF:ejection fraction, fQRSd: filtered QRS duration, RMS 40: root mean square 40 ms, LAS 40: under 40 uV duration, Data are presented as mean ± SD,

* p<0.05 between *SEMA3A* ^I334V (+)^ vs *SEMA3A* ^I334V(−)^.

Some of the patients with *SEMA3A* ^I334V^ had sinus bradycardia, and their RR intervals on the 12-lead ECG tended to be longer than those without (1031±111 ms vs. 932±182 ms, *P* = 0.039). None of the UCA subjects regularly took β-blockers during their ECG recordings. One patient without Sema3a^I334V^ took 100 mg/day of oral amiodarone when recording the ECG. The other cases did not have any anti-arrhythmic agents. Early repolarization (ER) was evident in only two *SEMA3A* ^I334V^ cases (15.4%), whereas 34 patients (48.6%) without *SEMA3A* ^I334V^ demonstrated ER (*P* = 0.02). The other 12-lead ECG parameters, signal-averaged ECG, and echocardiographic findings, were similar in the patients with and without *SEMA3A* ^I334V^.

### Screening of the *SEMA3A* region using tag SNPs

To screen the entire SEMA3A gene, 47 tag SNPs were additionally genotyped in the UCA patients from Hiroshima/Nagasaki University and the healthy controls from Hiroshima University (Table S1). All SNPs were successfully genotyped in >98% of the samples. Among them, one SNP, rs1533996, was not polymorphic. The other SNPs were within the Hardy-Weinberg equilibrium (P>0.01) in the controls except for rs13437857 (P = 0.0031) and rs10280701 (P = 0.000086). The p value of the I334V in the population (p = 4.53E-08) was still significant even if a Bonferroni correction for the tag-SNP approach was applied (p = 2.12E-06). None of the 47 tag SNPs were significantly associated with UCA after the Bonferroni correction. The I334V variant showed a moderate linkage disequilibrium only with rs740948 (r^2^ = 0.43). A haplotype analysis revealed that no haplotype had a stronger association with UCA than the single marker analysis (data not shown).

### Sympathetic nerve localization and nerve growth factor (NGF) expression in UCA patients with and without *SEMA3A* ^I334V^ (rs138694505)

**Figure 3:**
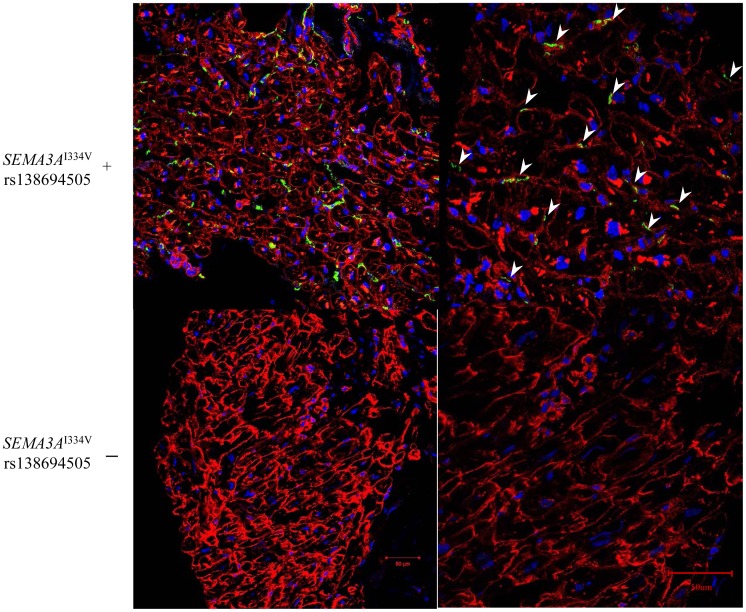
Immunofluorescence staining for Vinculin and anti-TH in the subendocardial layer of patients with and without *SEMA3A* ^I334V^. Immunofluorescence staining of cardiac biopsy specimens revealed that TH positive nerves, as sympathetic nerves, which are absent in the subendocardial layer in normal hearts, extended to the subendocardial layer only in patients with *SEMA3A* ^I334V^ (red; anti-Vinculin, green; anti-TH ). The samples were examined using a confocal microscope and captured with a 20×objective lens in the figures on the left and with a 40× objective lens in the figures on the right. The arrowheads show the TH positive nerves (Upper panels: *SEMA3A* ^I334V^+, Lower panels: *SEMA3A* ^I334V^−).

**Figure 4:**
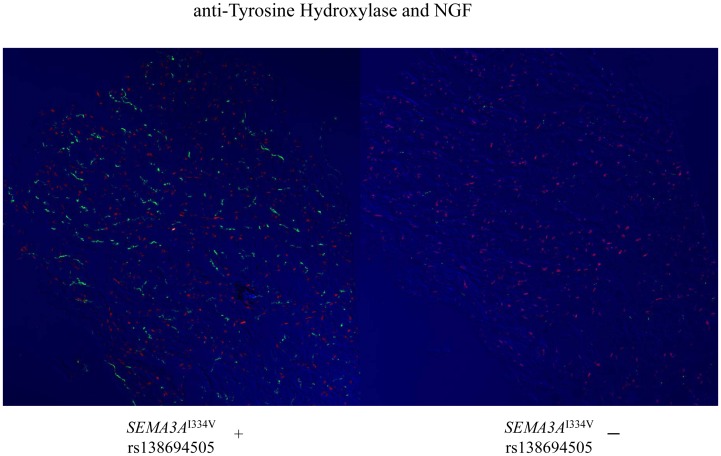
Immunofluorescence staining for Vinculin and NGF in the subendocardial layer of patients with and without *SEMA3A* ^I334V^. On the other hand, the levels of the NGF, a neural attractant factor, were expressed in the subendocardial layer and are comparable between patients with (left panel) and without (right panel) *SEMA3A* ^I334V^ (red; anti-NGF; green: anti-TH).

Representative immunofluorescence images for vinculin (a cell surface marker) and anti-tyrosine hydroxylase (TH) in the sympathetic nerves in the subendocardial layer of patients with and without *SEMA3A* ^I334V^ are shown in Figure 3. Under normal conditions, the TH nerves were reported to exist in the subepicardial layer of cardiomyocytes, not in the subendocardial layer (9). In patients without *SEMA3A* ^I334V^, no TH nerves were observed in the subendocardial layer, consistent with earlier findings in normal subjects. In patients with *SEMA3A* ^I334V^, in contrast, TH nerves were distributed in the subendocardial layer (right panel, the arrowheads indicate TH positive nerves). This finding was consistently observed in patients with *SEMA3A* ^I334V^ (N = 4) but not without *SEMA3A* ^I334V^ (N = 8), suggesting abnormal sympathetic innervation in the heart of UCA patients with SEMA3A^I334V^. On the other hand, NGF, a neural attractant factor, was similarly expressed in the subendocardial layer in patients with and without *SEMA3A* ^I334V^ (Figure 4).

### Expression and function of *SEMA3A* and *SEMA3A* ^I334V^

**Figure 5:**
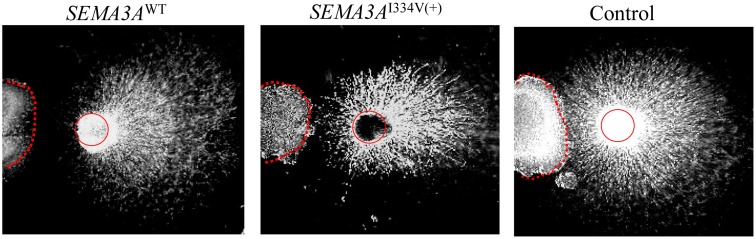
DRG repulsion assay of the *SEMA3A* ^WT^, *SEMA3A* ^I334V^, or control. *SEMA3A* ^WT^ expressing cells repelled DRG axons on the proximal side of the ganglia (left). In contrast, DRG explants were less responsive to *SEMA3A* ^I334V^ (middle).

As a result of a DRG repulsion assay, *SEMA3A* ^WT^-expressing cells repelled the DRG axons on the proximal side of the ganglia (Figure 5, left). In contrast, DRG explants were less responsive to *SEMA3A* ^I334V^ (Figure 5, middle).

**Figure 6:**
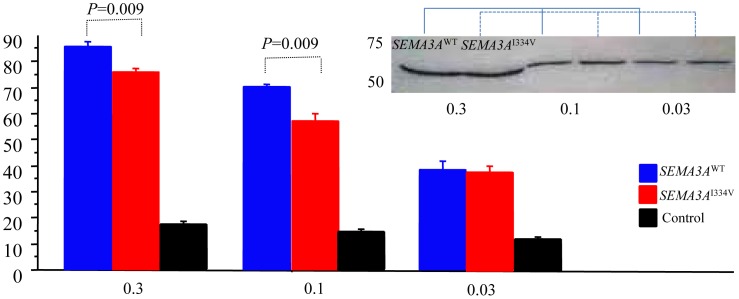
Growth cone collapse assay of the *SEMA3A* ^WT^, *SEMA3A* ^I334V^, or control. The percent of collapsed growth cones of the E8 chick embryos incubated with medium containing vector only, *SEMA3A* ^WT^, or *SEMA3A* ^I334V^ at dilutions (0.03, 0.1, and 0.3) of a concentrated media. All dilutions of the concentrated media of the *SEMA3A* or *SEMA3A* ^I334V^ expressed in HEK293T cells were similarly secreted. *SEMA3A* ^WT^ and *SEMA3A* ^I334V^ led to a collapse of the DRG neuron growth cones in all concentrations, but growth cone collapses by *SEMA3A* ^I334V^ (red bar) were significantly less than those by *SEMA3A* ^WT^ (blue bar) at the dilutions (0.3, 0.1) of the concentrated media (*P* = 0.009).

Figure 6 shows the percentage of collapsed growth cones in the E8 chick embryos incubated with media containing *SEMA3A* ^WT^, *SEMA3A* ^I334V^ and vector only (negative control) at 0.3, 0.1, and 0.03 dilutions of a concentrated media, respectively. At all dilutions, *SEMA3A* ^WT^, and *SEMA3A* ^I334V^ were similarly expressed and secreted (Figure 6). The secreted proteins for both *SEMA3A* ^WT^ and *SEMA3A* ^I334V^ were similar in size (approximately 65 kDa). The growth cone collapse by *SEMA3A* ^I334V^ was less frequent than that of *SEMA3A* ^WT^ at all concentrations. (*SEMA3A* ^WT^ vs. *SEMA3A* ^I334V^: 84.8±1.5% vs. 75.8±1.8% at a dilution of 0.3, *P* = 0.009, and 70.2±1.1% vs. 57.2±2.4% at a dilution of 0.1, *P* = 0.009; Figure 6, lower).

## Discussion

To the best of our knowledge, this is the first report demonstrating that UCA patients have a high incidence of I334V SNP (rs138694505) in the *SEMA3A* located at chromosome 5. Furthermore, new experimental data presented here indicates that *SEMA3A* ^I334V^ disrupts the *SEMA3A* function of inhibiting neural growth and impaired appropriate innervation patterning in the heart. Finally, this study suggested that *SEMA3A* ^I334V^ is a risk factor for human UCA and contributes to the pathogenesis of UCA.

Many studies have reported the relationship between abnormal autonomic nerve activity and lethal ventricular arrhythmias, and in most of them I^123^-MIBG imaging was used to aid in the detection of sympathetic innervation abnormalities [3]–[5], [11]. However, the molecular mechanisms determining these innervation densities in patients with lethal arrhythmia have not been fully clarified. Elucidation of underlying genetic defects will provide further insight into the pathogenesis of UCA, but identification of the genes involved in UCA is very difficult because of its high mortality rate and subsequent diagnostic difficulties. Unlike other monogenic arrhythmia syndromes (e.g., long QT syndrome, catecholaminergic polymorphic ventricular tachycardia and Brugada syndrome), the diagnosis of UCA cannot be made on the basis of ECG abnormalities prior to the occurrence of VF. In addition, UCA is only diagnosed by excluding any identifiable structural or functional cardiac diseases among the few survivors of VF. One case report indicated that a missense variant of the KCNJ8 gene, a subunit of the K_ATP_ channel, conferred a predisposition to dramatic depolarization changes and ventricular vulnerability [12]. In another report, Alders et al. demonstrated that a haplotype on chromosome 7, which includes the DPP6 gene (associated with potassium channel I_to_ subunits), was the causal gene of IVF [13], [14].

Sympathetic innervation of the heart is sculpted during development by chemoattractive factors such as NGF and chemorepulsive factors such as *SEMA3A*. NGF acts through the Trk A and p75 neurotrophin receptors in sympathetic neurons. Lorenz et al. reported heterogeneous ventricular sympathetic innervation, altered β-adrenergic receptor expression, and rhythm instability in mice lacking the p75 neurotrophin receptor within the heart [15]. Ieda et al. [9], [10] reported that cardiac innervation patterning is disrupted in *SEMA3A*-deficient and *SEMA3A*-overexpressing mice, leading to lethal arrhythmias and sudden death. On the basis of this background information, we focused on *SEMA3A*, which plays a crucial role in cardiac innervation patterning [7]–[10], [16], as abnormal sympathetic innervations have been demonstrated in patients with UCA. We observed that a polymorphism in exon 10 of the *SEMA3A* gene (i.e., *SEMA3A* ^I334V^), located in the semaphorin domain, which plays an essential role in *SEMA3A* [17], was highly prevalent in patients with UCA and strongly associated with UCA pathophysiology. To our knowledge, this is the first report that investigates the relevance of functional mutations or polymorphisms in *SEMA3A* with respect to human diseases.

We divided the case and control subjects into two geographical groups based on their birthplace in Japan. Significant results observed in Western Japan were replicated in the Eastern Japan group, and the combined P value and odds ratio calculated by the Mantel-Haenszel test were 0.0004 and 3.08, respectively.

According to publicly available data from the 1000 Genomes Project, the frequency of this risk allele of *SEMA3A* is similar among populations other than Europeans, suggesting that this variant may be relevant to the etiology of UCA across these populations. In our study, the G allele frequency was 2.8% in the controls, which was consistent with that reported in Japanese (3.9%) and East Asian populations (2.1%) in the 1000 Genomes Project.

Haïssaguerre et al. reported an increased prevalence of ER characterized by J-point elevation among patients with a history of UCA [18]. Antzelevitch et al. classified ER patterns for risk stratification of VF [19]. The genetic basis for ER is slowly coming into better focus. Burashnikow et al. identified loss of function mutations in the α1, β2, and α2δ subunits of the cardiac L-type calcium channels (CACNA1C, CACNB2, and CACNA2D1) in patients with ER syndrome [20]. Abe et al. reported that ER may be closely associated with depolarization abnormalities and autonomic modulation [21]. In this study, only two UCA cases with *SEMA3A* ^I334V^ demonstrated ER. Instead, the characteristics of the cases with *SEMA3A* ^I334V^ suffered VF attacks in a relaxed state and presented with sinus bradycardia/sinus node dysfunction. These findings are consistent with the report by Ieda et al. [9], [10] that *SEMA3A* ^−/−^ mice lacked a cardiac sympathetic innervation gradient and exhibited satellite ganglia malformations, which led to marked sinus bradycardia due to sympathetic dysfunction. Some of the UCA cases in our study may have a mild degree of depolarization or repolarization abnormalities, although we could not detect any obvious organic diseases such as cardiomiopathy by diagnostic imaging or manifest conduction disturbances. The other patients did not have any depolarization or repolarization abnormalities. The patients with *SEMA3A^I334V^* do not have a homogeneous phenotype and we have to follow up the clinical course of the UCA patients with *SEMA3A^I334V^* for a long period.

The frequency of AF was 21.6% and rather high in the UCA subjects of our study for unknown reasons and the frequency was similar in the patients with and without *SEMA3A^I334V^*. One possible reason was that the episodes of AF after resuscitation were included in the past history of AF.

In our study, immunofluorescence staining of the RV revealed that sympathetic nerves were distributed in the subendocardial layer only in patients with *SEMA3A* ^I334V^. If *SEMA3A* exists in adequate quantities in the endocardial layer and functions normally, sympathetic nerves extending to the endocardial layer are suppressed. We assumed that in UCA patients with *SEMA3A* ^I334V^, the epicardial-to-endocardial transmural sympathetic innervation patterning had deteriorated.

An *SEMA3A* ^WT^- and *SEMA3A* ^I334V^-concentrated media did not grossly affect the expression, stability, or secretion of the ligand. As for the molecular weight of *SEMA3A*, when it was expressed in HEK293, the full semaphorin domain (65 kDa) was cleaved and detected in a conditioned media [22]. The sizes of the secreted proteins in both *SEMA3A* ^WT^ and *SEMA3A* ^I334V^ were equal and coincident with the semaphorin domain including a dimerization interface and Neurolipin-1 (Nrp-1)-binding residue, and the biological activity was sufficient for the acquisition of a high repulsive activity [22].

The function of repelling the DRG axons was weaker and growth cone collapse was less frequent in *SEMA3A* ^I334V^ than in *SEMA3A* ^WT^. Therefore, one allele of *SEMA3A* leads to a disruption of the sympathetic innervation of the heart under relevant conditions. These findings were consistent with immunofluorescence observations strongly suggesting that *SEMA3A* ^I334V^ can disrupt the ability of *SEMA3A* to repel or collapse DRG axons and sensory neuron growth cones under equal conditions of the neural attractant NGF.

Merte et al. reported that a forward genetic screen in mice identified a novel loss of function *SEMA3A* ^K108N^ mutation, which bound to Nrp-1 but failed to repel or collapse DRG axons in vitro [23]. *SEMA3A* ^I334V^ exists in blade 5 of the 7-bladed propeller structure of the semaphorin domain and performs a crucial function in *SEMA3A*. Residues 333–335 in 5S of *SEMA3A* constitute the dimerization interface. The *SEMA3A*-65K dimerization interface overlaps with sites responsible for the initial high-affinity binding to the domain of Nrp-1. Binding of *SEMA3A* to Nrp-1 leads to a conformational change in Plexin-A1, which is transmitted to the cytosolic domain [17].

In the association analysis, *SEMA3A* ^I334V^ was highly prevalent in patients with UCA and associated with the UCA pathophysiology. On the other hand, none of the control subjects with *SEMA3A* ^I334V^ had any signs of disease at the time of the study, indicating incomplete penetrance or additional environmental or genetic factors.

Our study had several limitations. First, it was very difficult to congregate many UCA cases and therefore the size of our study population was too small to obtain any robust findings. Secondly, we were not able to study the segregation data in the UCA patients with SEMA3A^I334^ because their families refused screening. A future prospective study with a larger cohort will be required to obtain these data. A further functional study would also be desirable to determine whether any abnormal innervation can be observed in healthy carriers by using autopsy specimens

In conclusion, a polymorphism of *SEMA3A* ^I334V^ diminishes the cardiac sympathetic innervation gradient and partially contributes to the etiology of UCA. This finding is important in elucidating the pathogenesis of UCA.

## Materials and Methods

### Subjects

We recruited a total of 83 UCA patients (64 male and 19 female, mean age 43±16 years) from Hiroshima University Hospital, Nagasaki University Hospital, Shiga University of Medical Science, and the National Cerebral and Cardiovascular Center. We recruited 2958 controls (1540 male and 1452 female, mean age 54±18 years) from Hiroshima University Hospital, Osaka-Midosuji Rotary Club (Osaka, Japan), Shiga University of Medical Science, and Niigata University Graduate School of Medical and Dental Sciences. All patients and controls in this paper were unrelated Japanese individuals. Case and control subjects were collected from various regions of Japan. Although the Japanese population has rather low genetic diversity, it has been shown that population structures may lead to spurious associations [24]. Therefore, to eliminate the possibility of a population stratification, we divided case and control subjects into two groups geographically based on their birthplace information (i.e., Western Japan and Eastern Japan) (Figure S1).

The Institutional Ethics Committee of the Graduate School of Biomedical Science at Hiroshima University approved all procedures involving human tissue usage. Written informed consent was obtained from all subjects prior to participation.

Twelve subjects enrolled in the study were diagnosed and treated at the Hiroshima University Hospital; the other subjects were diagnosed and treated at other affiliated hospitals and their information was provided to us.

### Diagnosis of UCA

We defined UCA as that without structural heart disease and in the absence of signs of an arrhythmia syndrome such as Brugada syndrome, catecholaminergic polymorphic ventricular tachycardia and long QT syndrome. All patients with cardiac arrest underwent a physical examination, 12 lead ECG [25], echocardiography and coronary angiography to rule out any underlying heart disease. Those who met the inclusion criteria were enrolled and underwent additional testing (signal averaged ECG, T wave alternance, cardiac magnetic resonance imaging, computer tomography, provocation tests, cardiac biopsy or an electrophysiological study), if possible. The numbers of further noninvasive or invasive tests against UCA patients varied from institute to institute. Patients with exonic mutations in SCN5A and a positive pilsicainide challenge test were excluded from the sample. Early repolarization (ER) was defined as a QRS slurring or notching of ≥0.1 mV in more than two consecutive leads of the 12-lead ECG.

### Sequence analysis of *SEMA3A* genomic DNA and genotyping

Peripheral blood was obtained from all the subjects. Genomic DNA was extracted from leukocytes using a QIAamp DNA Blood Mini Kit (QIAGEN, Hilden, Germany) according to the standard protocol. Using Go Taq (Promega, Madison, WI, USA), all coding regions of the *SEMA3A* located at chromosome 5 were amplified by PCR from 2.5-ng genomic DNA using our original primers in 17 UCA patients and 15 healthy controls entered from Hiroshima University. These amplified coding regions were then resequenced using an ABI PRISM 310 Genetic Analyzer (Applied Biosystems, Foster City, CA, USA) to identify mutations and polymorphisms.

Subsequently, SNP genotypes were genotyped in All of the UCA subjects and healthy control subjects using the Invader assay or the TaqMan assay, as described previously [26], [27].

### Tag SNP selection

The 47 tag SNPs were genotyped only in the UCA patients and the healthy controls entered from Hiroshima University and Nagasaki University. Using the HapMap database (public release #27, hapmap.ncbi.nlm.nih.gov) and the Haploview program (www.broad.mit.edu/mpg/haploview) and based on selection criteria of r^2^>0.8 and a minor allele frequency of >0.01 for the Japanese population, tagging-SNPs were selected from the *SEMA3A* region spanning approximately 247 kb, from approximately 5 kb upstream of the transcription start site to 5 kb downstream of the 3′ untranslated region.

### Plasmid construction

The complete coding region of human *SEMA3A* was amplified from cDNA with forward (tgttagtgttgccatgaggtct) and reverse (gcattcacctgtgttctctgttag) primers. To generate Flag- *SEMA3A*, the coding sequence DYKDDDD was introduced between the codons for G25 and K26 (NM_006080.2). The I334V mutation was introduced by site-directed mutagenesis using the QuickChange (Stratagene, La Jolla, CA, USA). Full-length human wild-type (*SEMA3A* ^WT^) or mutant *SEMA3A* (*SEMA3A* ^I334V^) cDNA was cloned into pcDNA3.1(+) (Invitrogen, Carlsbad, CA, USA).

### Immunofluorescence staining of anti-tyrosine hydroxylase (TH), nerve growth factor (NGF), and vinculin

Transverse sections of a septal site of the RV outflow tract were obtained by biopsy from 12 UCA subjects (4 patients with *SEMA3A* ^I334V^ and 8 patients without *SEMA3A* ^I334V^). These sections were embedded in an OCT compound (Sakura, Torrance, CA, USA) and frozen with liquid nitrogen. Immunofluorescence staining was performed using the frozen sections with rabbit anti-TH (AB152, Millipore, Billerica, MA, USA) antibodies and mouse anti-vinculin (Sigma-Aldrich, St. Louis, MO, USA) antibodies diluted at concentrations of 1∶100 and 1∶200, respectively, in 1% BSA/PBS. Alexa 488-conjugated goat anti-rabbit and Alexa 568-conjugated goat anti-mouse antibodies (Invitrogen) were used as secondary antibodies. As for NGF, sheep polyclonal to NGF (ab49205, Abcam, Cambridge, MA, USA) and rabbit anti-TH (ab152, Millipore) were used as primary antibodies at concentrations of 1∶100 in 1% BSA/PBS. Alexa568 donkey anti-sheep (A21099) and Alexa488 donkey anti-rabbit (A21206) antibodies were used as secondary antibodies. Nuclei were stained with 10 µM of Hoechst 33342 (Molecular probes). Samples were examined using a confocal microscope and captured with a 20× and 40× objective lens on a Zeiss LSM 510 laser scanning microscopy system (Carl Zeiss, Thornwood, NY, USA).

### DRG repulsion assay and growth cone collapse assay of *SEMA3A* ^WT^ and *SEMA3A* ^I334V^

The DRG were dissected from E8 chick embryos. HEK293T cells were transfected with Flag- *SEMA3A* ^WT^ or *SEMA3A* ^I334V^ expression vector or equal amounts of empty vector (control) using Gene Juice Transfection Reagent (Novagen, Madison, WI, USA). The DRG and *SEMA3A* -expressing HEK293T cell aggregates were embedded as described previously [28]. Samples were incubated at 37°C in a 5% CO_2_ humidified incubator for 48 h and examined using an inverted microscope. For DRG repulsion assays, 10–15 DRG cells were examined, each with Sema3A^WT^, Sema3A^I334V^, or a control.

For the purpose of a growth cone collapse assay, the conditioned medium of the *SEMA3A* -expressing HEK293T cells was concentrated [22]. A Western blot analysis was performed using both dilutions of the *SEMA3A* ^WT^ and *SEMA3A* ^I334V^ concentrated media with anti-FlagM2 (Sigma). Growth cone collapse assays were performed as previously described using chick E8 DRG explants grown on laminin (Invitrogen)- and poly-L-lysine (Sigma)-coated 48-well plates (BD Falcon/353078). The dilution series of the *SEMA3A^WT^*, *SEMA3A^I334V^* and vector only concentrates were added to each well and incubated at 37°C in a 5% CO_2_ humidified incubator for 30 min. The explants were fixed with 4% paraformaldehyde in 10% sucrose PBS (pH 7.4), and the samples were examined using an inverted microscope [29]. In each dilution series, 5 or 6 growth cone collapse assays were investigated. Each in vitro assay was performed in triplicate.

For quantification, we counted at least 50 growth cones to score on each explant. We assigned each growth cone as either collapsed or not collapsed, and the results were expressed as the percentage of collapsed to all counted growth cones. We compared the percentage of those collapsed between the *SEMA3A* ^WT^ and *SEMA3A* ^I334V^.

### Statistical analysis

Normally distributed continuous variables are presented as the mean ± SD. Continuous data between the two groups were analyzed using the nonparametric Mann–Whitney U test. For testing the genetic associations in the case–control studies, the chi-square test and Cochran–Armitage trend test were used. Tests for the Hardy–Weinberg equilibrium among the cases and controls were conducted for observed and expected genotype frequencies using an ordinary chi-square test, where a *P*-value of <0.05 was considered statistically significant. For a meta-analysis of 3 individual cases and controls, we used the Mantel-Haenszel test.

## Supporting Information

Figure S1 The case and control subjects were divided into two groups geographically based on their birthplace information (i.e., Western Japan and Eastern Japan).
(PDF)

Table S1 Forty-seven tag SNPs of *SEMA3A* were additionally genotyped in the UCA patients and the healthy controls from Hiroshima University. The I334V variant had a moderate linkage disequilibrium only with rs740948 (r^2^ = 0.43). None of the other SNPs were significantly associated with the UCA after a Bonferroni correction. a: Tagging-SNPs other than I334V were selected based on the selection criteria of an r 2 of >0.8 and minor allele frequency of >0.01in the HapMap-JPT population. b: chi-square test P value in the allele frequency model (uncorrected). c: Hardy-Weinberg equilibrium tests in the control subjects.
(DOCX)

Table S2 The number of tests that we performed for the UCA in VF patients.
(DOCX)

Table S3 Phenotype characterizations in each UCA patient with *SEMA3A^I334V^*. Patient 3 had persistent AF and patient 13 had chronic AF. Patients 1,2,5,7 and 9 had 1st degree atrioventricular block. Patient 1 had positive late potentials and the fQRSd was increased in a number of patients.
(DOCX)
