# Supplementary figures and images for: A Nonsynonymous Polymorphism in Semaphorin 3A as a Risk Factor for Human Unexplained Cardiac Arrest with Documented Ventricular Fibrillation
Source: PLoS Genet. 2013 Apr 11;9(4):e1003364. doi: 10.1371/journal.pgen.1003364 (PMC3623806; doi:10.1371/journal.pgen.1003364)

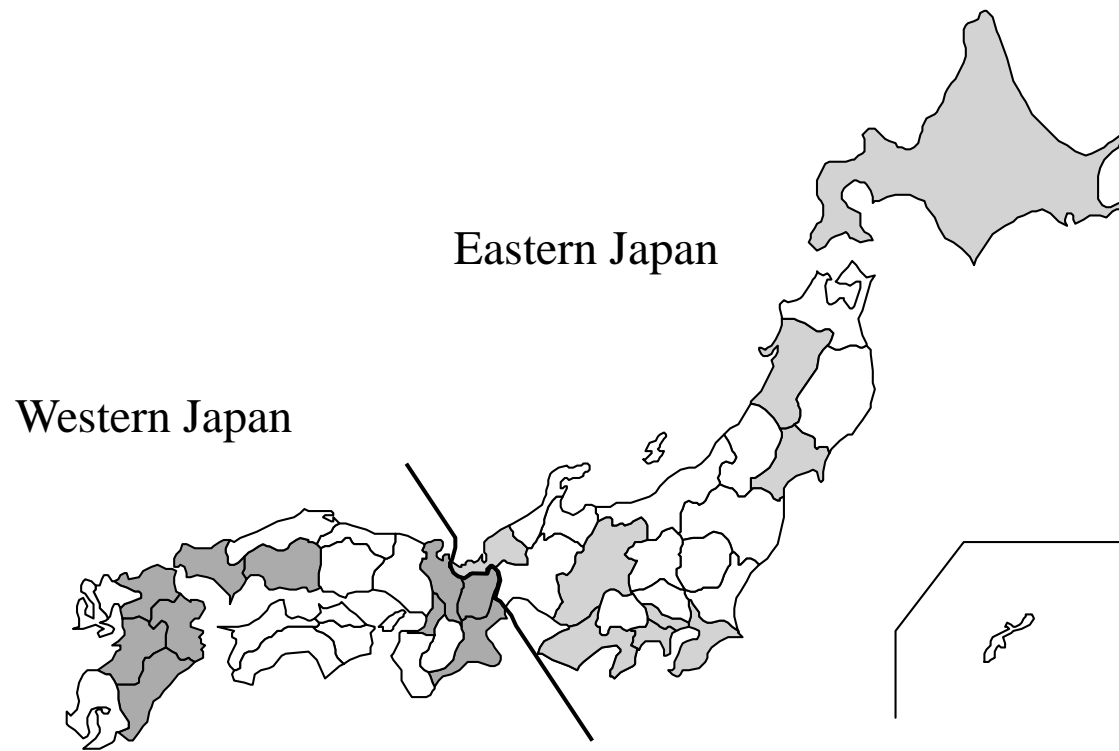

Supplement: Figure S1 — The case and control subjects were divided into two groups geographically based on their birthplace information (i.e., Western Japan and Eastern Japan). (PDF) [file pgen.1003364.s001.pdf]
