# Supplementary material for: A Nonsynonymous Polymorphism in Semaphorin 3A as a Risk Factor for Human Unexplained Cardiac Arrest with Documented Ventricular Fibrillation
Source: PLoS Genet. 2013 Apr 11;9(4):e1003364. doi: 10.1371/journal.pgen.1003364 (PMC3623806; doi:10.1371/journal.pgen.1003364)
Supplement: Table S1 — Forty-seven tag SNPs of SEMA3A were additionally genotyped in the UCA patients and the healthy controls from Hiroshima University. The I334V variant had a moderate linkage disequilibrium only with rs740948 (r2 = 0.43). None of the other SNPs were significantly associated with the UCA after a Bonferroni correction. a: Tagging-SNPs other than I334V were selected based on the selection criteria of an r 2 of >0.8 and minor allele frequency of >0.01in the HapMap-JPT population. b: chi-square test P value in the allele frequency model (uncorrected). c: Hardy-Weinberg equilibrium tests in the control subjects. (DOCX) [file pgen.1003364.s002.docx]

TableS1 The relationship of the SEMA3A genetic variants in UCA with VF

| SNP^a^ | Chr | position | region | Allele  1/2 | UCA with VF | | |  | Control | | | OR | (95%CI) | P value^b^ | HWE^c^ |
| --- | --- | --- | --- | --- | --- | --- | --- | --- | --- | --- | --- | --- | --- | --- | --- |
|  |  |  |  |  | 11 | 12 | 22 |  | 11 | 12 | 22 |  |  |  |  |
| rs12673519 | 7 | 83665936 | upstream | A/G | 1 | 17 | 21 |  | 87 | 446 | 592 | 0.85 | ( 0.51 - 1.4 ) | 0.53 | 0.81 |
| rs10256270 | 7 | 83659338 | intron 1 | A/G | 23 | 14 | 2 |  | 709 | 375 | 44 | 0.86 | ( 0.50 - 1.5 ) | 0.58 | 0.52 |
| rs11976488 | 7 | 83657451 | intron 1 | A/G | 30 | 9 | 0 |  | 800 | 301 | 27 | 1.43 | ( 0.71 - 2.9 ) | 0.32 | 0.83 |
| rs12707628 | 7 | 83655831 | intron 1 | C/T | 11 | 17 | 11 |  | 230 | 595 | 300 | 1.13 | ( 0.72 - 1.8 ) | 0.59 | 0.04 |
| rs17158709 | 7 | 83649125 | intron 1 | A/G | 27 | 12 | 0 |  | 786 | 312 | 29 | 1.08 | ( 0.58 - 2.0 ) | 0.81 | 0.77 |
| rs9642175 | 7 | 83640887 | intron 1 | C/T | 17 | 17 | 5 |  | 503 | 498 | 121 | 0.93 | ( 0.58 - 1.5 ) | 0.76 | 0.89 |
| rs12666837 | 7 | 83639293 | intron 1 | A/G | 0 | 9 | 30 |  | 29 | 282 | 816 | 0.73 | ( 0.36 - 1.5 ) | 0.39 | 0.44 |
| rs11974667 | 7 | 83634556 | intron 1 | A/C | 0 | 3 | 36 |  | 0 | 51 | 1077 | 1.73 | ( 0.53 - 5.7 ) | 0.43 | 0.44 |
| rs6966352 | 7 | 83628698 | intron 1 | C/T | 0 | 3 | 36 |  | 2 | 64 | 1061 | 1.29 | ( 0.40 - 4.2 ) | 0.51 | 0.32 |
| rs7789995 | 7 | 83627456 | intron 1 | A/T | 35 | 3 | 0 |  | 1012 | 112 | 2 | 1.32 | ( 0.41 - 4.3 ) | 1.00 | 0.55 |
| rs13437857 | 7 | 83624628 | intron 1 | A/G | 24 | 13 | 2 |  | 690 | 405 | 32 | 0.94 | ( 0.55 - 1.6 ) | 0.83 | 0.0031 |
| rs447 | 7 | 83610351 | intron 1 | A/G | 0 | 5 | 34 |  | 5 | 120 | 997 | 1.11 | ( 0.44 - 2.8 ) | 0.82 | 0.50 |
| rs10488270 | 7 | 83608284 | intron 1 | A/C | 2 | 9 | 27 |  | 37 | 326 | 762 | 0.95 | ( 0.52 - 1.8 ) | 0.88 | 0.77 |
| rs3801655 | 7 | 83591271 | intron 3 | G/C | 0 | 3 | 36 |  | 2 | 116 | 1010 | 0.71 | ( 0.22 - 2.3 ) | 0.80 | 0.48 |
| rs12532852 | 7 | 83590819 | intron 3 | A/C | 38 | 1 | 0 |  | 1091 | 27 | 0 | 0.94 | ( 0.13 - 7.0 ) | 0.62 | 0.68 |
| rs1990046 | 7 | 83585578 | intron 3 | A/G | 0 | 0 | 39 |  | 0 | 23 | 1104 |  |  | 1.00 | 0.73 |
| rs929334 | 7 | 83582309 | intron 3 | C/T | 36 | 3 | 0 |  | 988 | 136 | 3 | 1.68 | ( 0.52 - 5.4 ) | 0.48 | 0.46 |
| rs2372032 | 7 | 83580426 | intron 3 | G/T | 26 | 10 | 3 |  | 646 | 420 | 62 | 1.23 | ( 0.71 - 2.2 ) | 0.46 | 0.56 |
| rs727650 | 7 | 83573774 | intron 4 | A/G | 9 | 15 | 15 |  | 241 | 565 | 322 | 0.85 | ( 0.54 - 1.3 ) | 0.48 | 0.82 |
| rs3801631 | 7 | 83572925 | intron 4 | A/G | 3 | 12 | 24 |  | 60 | 399 | 666 | 1.00 | ( 0.59 - 1.7 ) | 1.00 | 0.98 |
| rs3801629 | 7 | 83572529 | intron 4 | A/G | 12 | 16 | 11 |  | 267 | 584 | 275 | 1.07 | ( 0.68 - 1.7 ) | 0.78 | 0.21 |
| rs7801391 | 7 | 83571857 | intron 4 | A/C | 18 | 15 | 6 |  | 482 | 514 | 130 | 0.99 | ( 0.62 - 1.6 ) | 0.96 | 0.69 |
| rs7801600 | 7 | 83564541 | intron 4 | C/T | 30 | 8 | 1 |  | 878 | 226 | 21 | 0.92 | ( 0.47 - 1.8 ) | 0.81 | 0.15 |
| rs7808864 | 7 | 83561283 | intron 4 | A/G | 0 | 6 | 33 |  | 13 | 221 | 892 | 0.68 | ( 0.29 - 1.6 ) | 0.36 | 0.87 |
| rs2691694 | 7 | 83540412 | intron 4 | A/G | 0 | 2 | 37 |  | 2 | 93 | 1031 | 0.58 | ( 0.14 - 2.4 ) | 0.77 | 0.95 |
| rs2372029 | 7 | 83538621 | intron 4 | A/G | 35 | 4 | 0 |  | 995 | 128 | 5 | 1.21 | ( 0.43 - 3.3 ) | 1.00 | 0.69 |
| rs17296151 | 7 | 83537669 | intron 4 | A/G | 33 | 5 | 0 |  | 912 | 204 | 12 | 1.60 | ( 0.64 - 4.0 ) | 0.31 | 0.88 |
| rs2527031 | 7 | 83536113 | intron 4 | C/T | 0 | 10 | 29 |  | 16 | 216 | 895 | 1.19 | ( 0.61 - 2.3 ) | 0.61 | 0.47 |
| rs2189849 | 7 | 83529507 | intron 4 | A/C | 8 | 19 | 12 |  | 185 | 548 | 394 | 1.18 | ( 0.75 - 1.9 ) | 0.46 | 0.81 |
| rs2527039 | 7 | 83527550 | intron 5 | C/T | 28 | 10 | 1 |  | 828 | 268 | 31 | 0.94 | ( 0.50 - 1.8 ) | 0.86 | 0.10 |
| rs12112784 | 7 | 83509137 | intron 6 | A/T | 6 | 15 | 18 |  | 92 | 483 | 553 | 1.26 | ( 0.78 - 2.0 ) | 0.34 | 0.35 |
| rs1533995 | 7 | 83502084 | intron 6 | A/G | 0 | 8 | 31 |  | 13 | 207 | 908 | 0.99 | ( 0.47 - 2.1 ) | 0.98 | 0.76 |
| rs17158533 | 7 | 83499275 | intron 6 | C/T | 4 | 22 | 13 |  | 203 | 546 | 365 | 0.84 | ( 0.53 - 1.3 ) | 0.45 | 0.96 |
| rs17158529 | 7 | 83497513 | intron 6 | C/T | 25 | 12 | 2 |  | 652 | 428 | 48 | 1.17 | ( 0.67 - 2.0 ) | 0.58 | 0.03 |
| rs1357814 | 7 | 83496605 | intron 6 | A/G | 1 | 11 | 27 |  | 56 | 344 | 721 | 0.78 | ( 0.43 - 1.4 ) | 0.43 | 0.076 |
| rs6976211 | 7 | 83489790 | intron 6 | C/T | 0 | 10 | 29 |  | 25 | 269 | 834 | 0.89 | ( 0.46 - 1.8 ) | 0.74 | 0.55 |
| rs6949238 | 7 | 83484752 | intron 6 | C/T | 17 | 21 | 1 |  | 543 | 477 | 107 | 1.06 | ( 0.65 - 1.7 ) | 0.83 | 0.88 |
| rs17158484 | 7 | 83476754 | intron 9 | A/C | 0 | 1 | 37 |  | 3 | 79 | 1045 | 0.34 | ( 0.047 - 2.5 ) | 0.53 | 0.25 |
| rs138694505 | 7 | 83474745 | exon 10 I334V | A/G | 29 | 10 | 0 |  | 1070 | 55 | 0 | 0.17 | ( 0.083 - 0.35 ) | 4.54E-08 | 0.40 |
| rs1533996 | 7 | 83474559 | intron 10 | C/G | 0 | 0 | 39 |  | 0 | 0 | 1128 |  |  |  |  |
| rs6944512 | 7 | 83470936 | intron 11 | A/G | 1 | 11 | 27 |  | 38 | 296 | 793 | 1.01 | ( 0.55 - 1.8) | 0.97 | 0.11 |
| rs3801600 | 7 | 83470530 | intron 11 | C/T | 0 | 10 | 29 |  | 17 | 227 | 881 | 1.12 | ( 0.57 - 2.2 ) | 0.74 | 0.59 |
| rs3801593 | 7 | 83467378 | intron 12 | C/T | 11 | 18 | 10 |  | 199 | 551 | 370 | 1.43 | ( 0.91 - 2.3 ) | 0.12 | 0.80 |
| rs10280701 | 7 | 83465783 | intron 12 | C/T | 39 | 0 | 0 |  | 1099 | 27 | 2 |  |  | 0.62 | 8.6E-05 |
| rs10487865 | 7 | 83448662 | exon 14 synonymous | C/G | 20 | 17 | 2 |  | 613 | 448 | 66 | 0.94 | ( 0.57 - 1.57 ) | 0.81 | 0.18 |
| rs740948 | 7 | 83437271 | intron 15 | C/T | 31 | 7 | 1 |  | 1016 | 86 | 3 | 0.33 | ( 0.16 - 0.69 ) | 0.0018 | 0.41 |
| rs797826 | 7 | 83422677 | downstream | T/C | 0 | 7 | 32 |  | 9 | 200 | 899 | 0.90 | ( 0.41 - 2.0 ) | 0.80 | 0.56 |
| rs797827 | 7 | 83421693 | downstream | A/G | 3 | 17 | 11 |  | 132 | 533 | 455 | 1.07 | ( 0.63 - 1.8 ) | 0.81 | 0.20 |

SEMA3A: Semaphorin 3A, UCA: unexplained cardiac arrest, VF:ventricular fibrillation

a: Tagging-SNPs other than I334V were selected based on the selection criteria of r 2 > 0.8 and minor allele frequency > 0.01in the HapMap-JPT population.

b: chi-square test P value in allele frequency model (uncorrected)

c: Hardy-Weinberg equilibrium tests in control subjects
