# Supplementary material for: A Nonsynonymous Polymorphism in Semaphorin 3A as a Risk Factor for Human Unexplained Cardiac Arrest with Documented Ventricular Fibrillation
Source: PLoS Genet. 2013 Apr 11;9(4):e1003364. doi: 10.1371/journal.pgen.1003364 (PMC3623806; doi:10.1371/journal.pgen.1003364)
Supplement: Table S2 — The number of tests that we performed for the UCA in VF patients. (DOCX) [file pgen.1003364.s003.docx]

Table S2.Number of tests that were performed for UCA with VF patients

|  | Number of performed tests | |
| --- | --- | --- |
|  | ***SEMA3A*^I334V^: rs138694505 (+)** | ***SEMA3A*^I334V^: rs138694505 (-)** |
| **Electrocardiography** |  |  |
| 12 leads ECG | 13 | 70 |
| Signal Averaged ECG | 8 | 35 |
| T wave alternance | 6 | 12 |
| Holter ECG | 6 | 15 |
| **Imaging** |  |  |
| Echocardiography | 13 | 70 |
| Coronary angiography | 13 | 70 |
| Cardiac CT | 6 | 38 |
| Cardiac MRI | 3 | 6 |
| **Provocation** |  |  |
| Pilsicainide | 11 | 53 |
| Epinephrine | 4 | 5 |
| **Discretionary** |  |  |
| electrophysiology | 3 | 20 |
| ventricular biopsy | 4 | 8 |

VF:ventricular fibrillation, UCA: unexplained cardiac arrest
