# Supplementary material for: A Nonsynonymous Polymorphism in Semaphorin 3A as a Risk Factor for Human Unexplained Cardiac Arrest with Documented Ventricular Fibrillation
Source: PLoS Genet. 2013 Apr 11;9(4):e1003364. doi: 10.1371/journal.pgen.1003364 (PMC3623806; doi:10.1371/journal.pgen.1003364)
Supplement: Table S3 — Phenotype characterizations in each UCA patient with SEMA3AI334V. Patient 3 had persistent AF and patient 13 had chronic AF. Patients 1,2,5,7 and 9 had 1st degree atrioventricular block. Patient 1 had positive late potentials and the fQRSd was increased in a number of patients. (DOCX) [file pgen.1003364.s004.docx]

TableS3. Phenotype characterizations in each UCA patints with SEMA3AI334V: rs138694505

| Patients | 1 | 2 | 3 | 4 | 5 | 6 | 7 | 8 | 9 | 10 | 11 | 12 | 13 |
| --- | --- | --- | --- | --- | --- | --- | --- | --- | --- | --- | --- | --- | --- |
| Age | 22 | 24 | 73 | 77 | 49 | 40 | 61 | 62 | 38 | 45 | 34 | 43 | 62 |
| Gender | male | female | female | male | male | female | male | male | male | male | male | male | female |
| Documented VF | + | + | + | + | + | + | + | + | + | + | + | + | + |
| History of syncope | - | + | + | - | + | - | - | + | - | + | - | - | - |
| History of atrial fibrillation | - | - | + | - | - | - | - | - | - | - | - | - | - |
| Family history of sudden cardiac death | + Father (40y) | +Mother (30y) | - | - | + | - | - | - | - | + | - | - | + |
| Time of VF events (20:00-8:00) | Yes | Yes | Yes | Yes | No | Yes | No | Yes | Yes | Yes | No | No | Yes |
| Situation at VF events | just woke up on bed | Relaxed at home | Relaxed at home | During sleeping | During a nap | During speaking | During exercise | During sleeping | just after woke up | During sleeping | During travel to work | During working | just after woke up |
| **Twelve Leads ECG Findings** |  |  |  |  |  |  |  |  |  |  |  |  |  |
| RR (ms) | 1060 | 1000 | 1120 | 1000 | 1090 | 1100 | 1080 | 850 | 1080 | 1000 | 1020 | 1220 | 1000 |
| PQ (ms) | 240 | 200 | 120 | 171 | 240 | 180 | 200 | 194 | 220 | 180 | 180 | 130 | AF |
| QRS (ms) | 240 | 80 | 100 | 97 | 100 | 68 | 96 | 90 | 140 | 120 | 120 | 100 | 80 |
| QTc | 430 | 430 | 480 | 470 | 430 | 360 | 427 | 456 | 430 | 420 | 406 | 424 | 400 |
| Presence of J wave | none | none | none | none | none | none | yes | none | none | none | none | none | yes |
| **Signal Averaged ECG findings** |  |  |  |  |  |  |  |  |  |  |  |  |  |
| fQRSd (ms) | 163 | 151 | 164 | 152 |  | 108 | 89 | not done | not done | not done | 101 | not done | 98 |
| RMS 40 (uV) | 7.3 | 39.7 | 24.2 | 27.1 |  | 13 | 93 |  |  |  | 43 |  | 68 |
| LAS 40 (ms) | 52 | 33 | 35 | 31 |  | 40 | 29 |  |  |  | 34 |  | 39 |
| **EPS Findings** |  |  |  |  | not done | not done | not done | not done | not done | not done | not done | not done |  |
| AH (ms) | 80 | 138 | AF | 99 |  |  |  |  |  |  |  |  | AF |
| HV (ms) | 41 | 46 |  | 41 |  |  |  |  |  |  |  |  |  |
| AVN ERP | 530 | 480 |  | 400 |  |  |  |  |  |  |  |  |  |
| %SRT | 150.1 | 155.7 |  | 120.6 |  |  |  |  |  |  |  |  |  |
| **Echocardiography findings** |  |  |  |  |  |  |  |  |  |  |  |  |  |
| LVDd (mm) | 52 | 48 | 52 | 39 | 47 | 46 | 41 | 38 | 45 | 49 | 50 | 44 | 52 |
| IVSTd (mm) | 8 | 8 | 9 | 9 | 11 | 11 | 9 | 7 | 10 | 9 | 9 | 8 | 9 |
| EF (%) | 66 | 60 | 53 | 66 | 73 | 79 | 68 | 72 | 59 | 64 | 65 | 65 | 70 |

VF:ventricular fibrillation, SEMA3A: Semaphorin 3A,UCA: unexplained cardiac arrest

fQRSd: filtered QRS duration, RMS 40: root mean square 40 ms, LAS 40: under 40uV duration

LVDd: left ventricular end diastolic volume, IVSTd: interventricular septum thickness, EF:ejection fraction
